# Tyrosine Phosphatase Shp2 Mediates the Estrogen Biological Action in Breast Cancer via Interaction with the Estrogen Extranuclear Receptor

**DOI:** 10.1371/journal.pone.0102847

**Published:** 2014-07-21

**Authors:** Jun Li, Yujia Kang, Longgang Wei, Wenjie Liu, Yingpu Tian, Baozhen Chen, Xiandong Lin, Yang Li, Gen-Sheng Feng, Zhongxian Lu

**Affiliations:** 1 Xiamen City Key Lab of Metabolism Disease & School of Pharmaceutical Sciences, Xiamen University, Xiamen, Fujian, China; 2 Department of Pathology, Fujian Provincial Tumor Hospital, Fuzhou, Fujian, China; 3 Department of Pathology & Division of Biological Sciences, University of California San Diego, La Jolla, California, United States of America; State Key Laboratory of Reproductive Biology, Institute of Zoology, Chinese Academy of Sciences, China

## Abstract

The extranuclear estrogen receptor pathway opens up novel perspectives in many physiological and pathological processes, especially in breast carcinogenesis. However, its function and mechanisms are not fully understood. Herein we present data identifying Shp2, a SH2-containing tyrosine phosphatase, as a critical component of extranuclear ER pathway in breast cancer. The research checked that the effect of Shp2 on the tumor formation and growth in animal model and investigated the regulation of Shp2 on the bio-effect and signaling transduction of estrogen in breast cancer cell lines. The results showed that Shp2 was highly expressed in more than 60% of total 151 breast cancer cases. The inhibition of Shp2 activity by PHPS1 (a Shp2 inhibitor) delayed the development of dimethylbenz(a)anthracene (DMBA)-induced tumors in the rat mammary gland and also blocked tumor formation in MMTV-pyvt transgenic mice. Estradiol (E2) stimulated protein expression and phosphorylation of Shp2, and induced Shp2 binding to ERα and IGF-1R around the membrane to facilitate the phosphorylation of Erk and Akt in breast cancer cells MCF7. Shp2 was also involved in several biological effects of the extranuclear ER-initiated pathway in breast cancer cells. Specific inhibitors (phps1, phps4 and NSC87877) or small interference RNAs (siRNA) of Shp2 remarkably suppressed E2-induced gene transcription (Cyclin D1 and trefoil factor 1 (TFF1)), rapid DNA synthesis and late effects on cell growth. These results introduced a new mechanism for Shp2 oncogenic action and shed new light on extranuclear ER-initiated action in breast tumorigenesis by identifying a novel associated protein, Shp2, for extranuclear ER pathway, which might benefit the therapy of breast cancer.

## Introduction

Recently, an increasing number of studies have found that estrogen can exert its action through a extranuclear estrogen receptor (ER) pathway [1], [2], which is thought to be required for the estrogen rapid signal, which triggers cytoplasmic kinase cascades to regulate other signals or activate transcriptional factors. The extranuclear ER pathway is involved in several crucial cellular functions such as cell proliferation, migration, secretion, and apoptosis [3], [4]. Knowledge on these novel estrogen actions is now significantly broadening our understanding of breast carcinogenesis, particularly regarding metastasis and drug resistance [5], [6]. However, mechanisms underlying rapid extranuclear responses of estrogen signal are not yet fully understood [6], [7].

The extranuclear estrogen receptor includes membrane-associated receptors (such as GPR30/GPER1) and cytoplasmic receptor [8]. Because estrogen receptor (ER) has no intrinsic transmembrane domain and/or kinase domain, the cytoplasmic ER requires association-proteins to translocate it to the plasma membrane and trigger the cytoplasmic pathway. Mounting evidences now suggest that a pool of intracellular receptors (IGF-1R and EGFR) and membrane receptor adapter proteins (G-protein, Shc, Src, p85α, and so on) are associated with the cytoplasmic ER signal pathway [9]–[11]. These associated proteins activate several cytoplasmic cascades, including PLC-PKC [12], [13], Ras-Raf-MAPK [14], [15], Src-PI3K-AKT [16], and cAMP-PKA [17]. Downstream pathways then lead to diversified cell type-specific estrogen actions, such as the triggering of the Ras-Raf-MAPK pathway in epithelial cells [15], Src-AKT-eNOS pathway in endothelial cells [18], or the PLC-cAMP-PKA pathway in neurons and intestinal cells. Therefore, the different expression patterns of these three party proteins are viewed as key factors in response to multiform and cell type-specific estrogen actions.

Tyrosine phosphatase protein Shp2 is a ubiquitously expressed and multifunctional protein [19], [20]. It consists of two Src homology 2 (SH2) domains and a protein tyrosine phosphatase (PTP) domain [19], [20]. Shp2 is induced to bind with the tyrosine residues of an phosphorylated protein (such as growth factor receptors) by two SH2 domains, and then dephosphorylates this protein activity with PTP domain [20]. But, the PTP activity of Shp2 is now believed to be required for the activation of several cytoplasmic protein kinases, such as Ras-raf-MAPK, PI3K-AKT and cAMP-PKA [19], [20]. By promoting the activation of these kinase proteins, Shp2 positively regulates cell growth and differentiation, organ development, immunological reaction, as well as metabolism. Shp2 is also involved in numerous diseases [21], [22], especially cancer [23], [24]. The human Shp2 gene, ptpn11, is regarded as the first proto-oncogene in the PTP family [23]. Its activated mutations are found in around 35% of sporadic juvenile myelomonocytic leukemia cases [25], and also in sporadic cases of several solid tumors such as those in the lung [26], [27], colon [28], [29], liver [30], and brain [31]. However, reports on the biological role of Shp2 in solid tumors are not compatible. Shp2 is overexpressed in gastric cancer and promotes tumor development [32], [33]; But, deletion of Shp2 in liver induces tumor formation in the mouse model [34].

Shp2, in cooperation with GRB2-associated binding protein 2 (Gab2), increases the proliferation of human breast epithelial MCF10A cells and enhances the metastasis of Her2/Neu-induced breast tumors in the transgenic mouse model [35]–[37]. Recently, Shp2 deletion has been found to block the growth and invasion of MCF10A cells in three-dimensional cultures, as well as reduces the metastasis of an established breast tumor in a xenograft mouse model [38]. However, the mechanism of Shp2 action in breast cancer cells is not yet clear. The Shp2 expression pattern is correlated with that of the estrogen receptors (ERs) in breast tumors [39], and the expression of Gab2, a pattern protein of Shp2, is induced by E2 in breast cancer cells [40]. Therefore, these results allowed us to hypothesize that Shp2 may be expressed in response to an estrogen signal and mediate the estrogen action associated with breast cancer.

The present research investigated that the effect of Shp2 on the growth of DMBA-induced tumor in the rat mammary gland and the tumor formation in MMTV-pyvt transgenic mice, and studied the regulation of Shp2 on the bio-effect and signaling transduction of estrogen in breast cancer cell lines. These results provide novel insight on the extranuclear ER pathway, and a new mechanism for Shp2 oncogenic action.

## Results

### Shp2 was overexpressed in breast tumor

**Figure 1:**
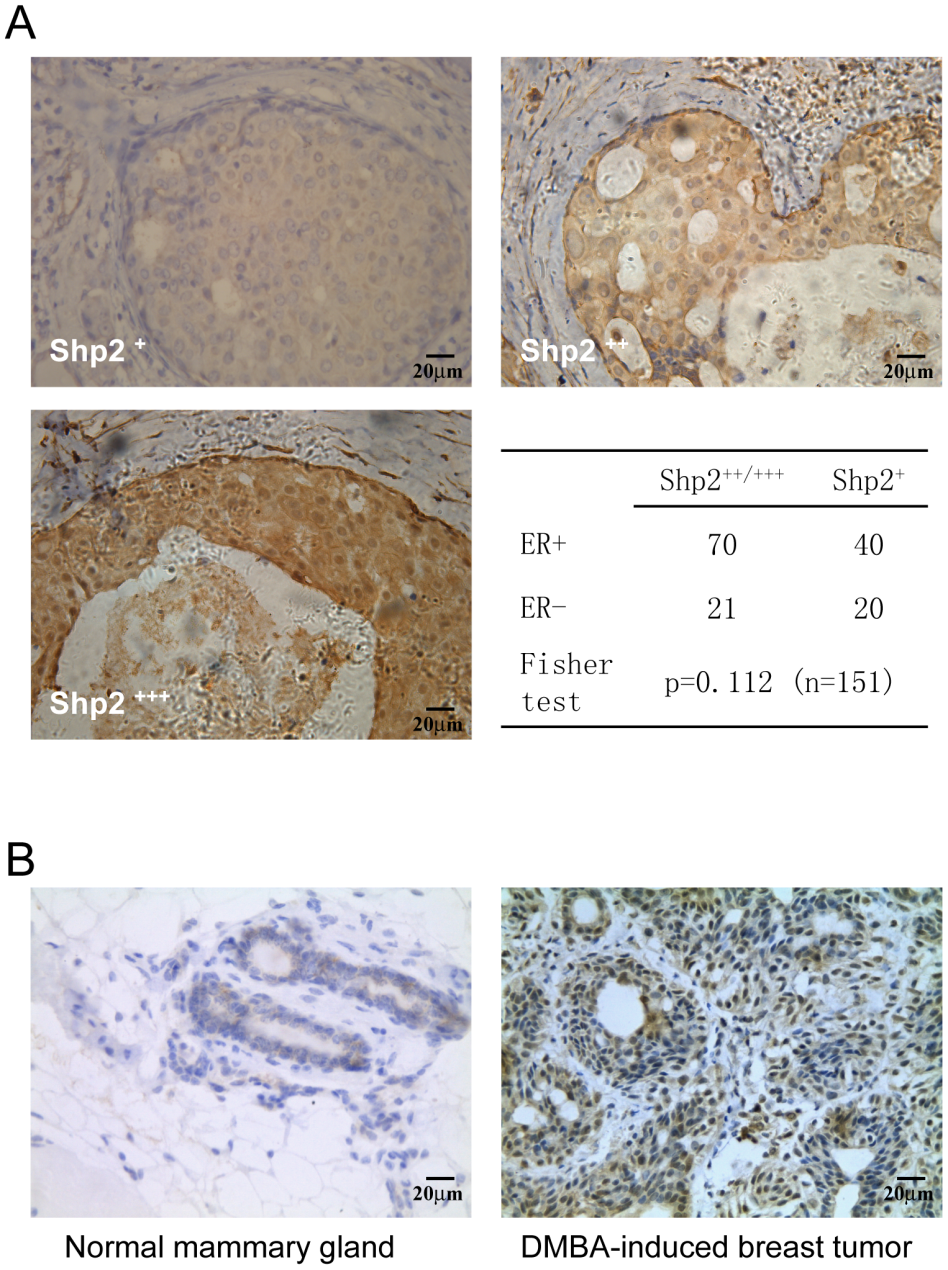
Shp2 was overexpressed in breast tumor. A: Pictures show the protein expression level of Shp2 in breast cancer samples, which were determined by immunostaining with anti-Shp2 antibody. Cells expressed Shp2 were stained in brown. (+) means a mild expression of Shp2, while ++ or +++ represents a strong expression of Shp2.The table shows that the Shp2 expression may be positively correlated with the expression of ERα with a lower p value (0.112, Fisher test) in the total 151 breast cancer cases. B: Shp2 protein was stained with Shp2 antibody in normal or DMBA–induced rat mammary gland tumor. Cells expressed Shp2 were stained in brown.

To view the expression pattern of Shp2 and its effects on the clinical progress of primary breast cancer, Shp2 was immunohistochemical stained with a specific antibody. Fig 1A shows different staining levels of Shp2 with brown color. Shp2 protein was observed in nearly all 151 samples, but had high expression (Shp2++ and Shp2+++) in 91 samples (more than 60% of the total 151) (Fig 1A table). We checked the relationship between high expression of Shp2 (Shp2++ or Shp2+++) and clinical or pathological progress, and found that the Shp2 expression level had no clear relation to any clinical or pathological progress indicator (such as grade, ERα expression (ER+), etc.) (Table S1). However, Shp2 still had a tendency to be highly expressed in a breast cancer sample with ER+ (p = 0.112, Fisher test) (Fig 1A, table). We also examined the expression of Shp2 in DMBA-induced tumor in rat mammary glands and discovered that high levels of Shp2 in the tumor (Fig 1B, right panel) compared with normal tissue (Fig 1B, left panel). These results showed that the expression of Shp2 protein was increased in breast tumor tissue, and suggested that Shp2 may play a role in tumorigenesis of breast tissue.

### Suppression of Shp2 activity blocked the tumor development in rodent mammary glands

**Figure 2:**
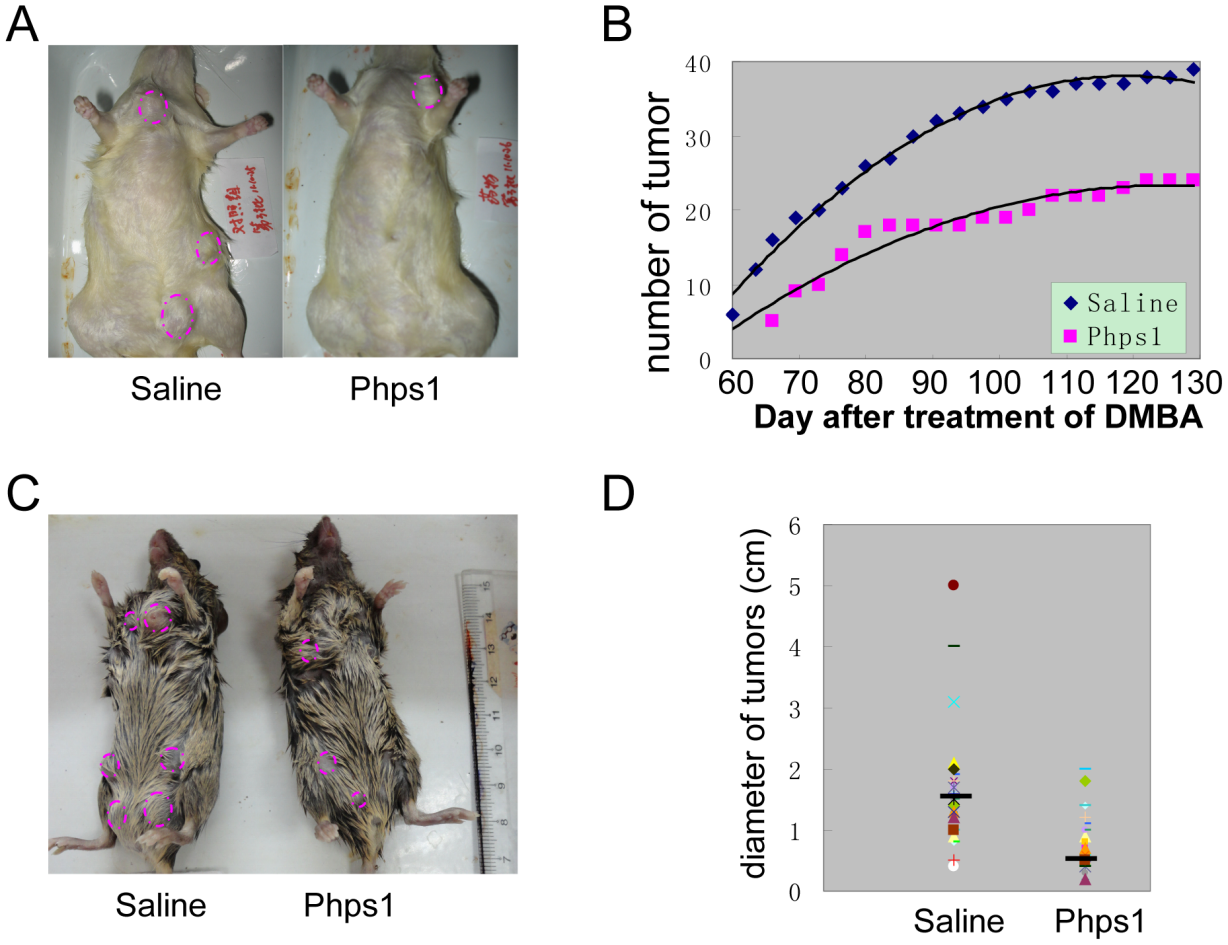
Suppressions of Shp2 activity blocked the tumor development in rodent mammary gland. A: The number and size of most of tumor in rats injected with phps1 (a specific inhibitor of Shp2 activity) were smaller than those in rats with saline. Each red circle represents a tumor. B: Rats were treated with DMBA to induce tumor formation in mammary glands, and then injected with the Shp2 activity inhibitor phps1. The number of tumors was counted at each observation day. Spots indicate the total number of tumors by injecting with saline (blue triangle) or phps1 (red square) at different days after DMBA treatment. C: phps1 treatment also decreased the number and size of tumors in MMTV-pyvt transgenic mice compared with control mice. Tumors were labeled with a red circle. D: Most tumors in mice injected with phps1 were smaller than those in control mice. Spots show the longer tumor diameter. The black bar is an average value.

To evaluate the biological role of Shp2 in breast tumorigenesis, we first checked the effect of the specific activity inhibitor of Shp2 on DMBA-induced mammary gland tumor development progress. In a typical procedure, 5∼6 week-old female rats were fed with DMBA, and then treated with Shp2 inhibitor phps1 four weeks later. After 6 weeks of DMBA treatment, tumors in the rat mammary gland were analyzed. In three repeated experiments, the first tumor was observed at day 53, 63 and 60 respectively after DMBA treatment in normal rats (control group). But, tumor formation was delayed and firstly showed at day 72, 80 and 66 respectively in the phps1-treated rats. Furthermore, tumors in each rat in each 3 day were successively observed in more than two months. At each observation time, the phps1-treated rats also had fewer tumors than the rat in the control group (Fig 2A, red circle). During the tumor growth term, the total number of tumors in the phps1 treatment group was always less than that in the control group at each observation day (Fig 2B). These observations indicated that phps1 may delay DMBA-induced breast tumorigenesis.

To support this result, 6 weeks old female MMTV-pyvt transgenic mice were treated with phps1 and their mammary gland tumors were examined. Phps1 exerted an inhibitory effect on tumor growth in transgenic mice. Compared with control mice, the number of tumor in each phps1-treated mouse was fewer (Fig 2C) and the tumor size was also smaller. The average tumor diameter of in phps1-treated mice was remarkably shorter than that in the control mice (Fig 2D).

### Estradiol upregulated the expression and phosphorylation of Shp2 in breast cancer cell lines

**Figure 3:**
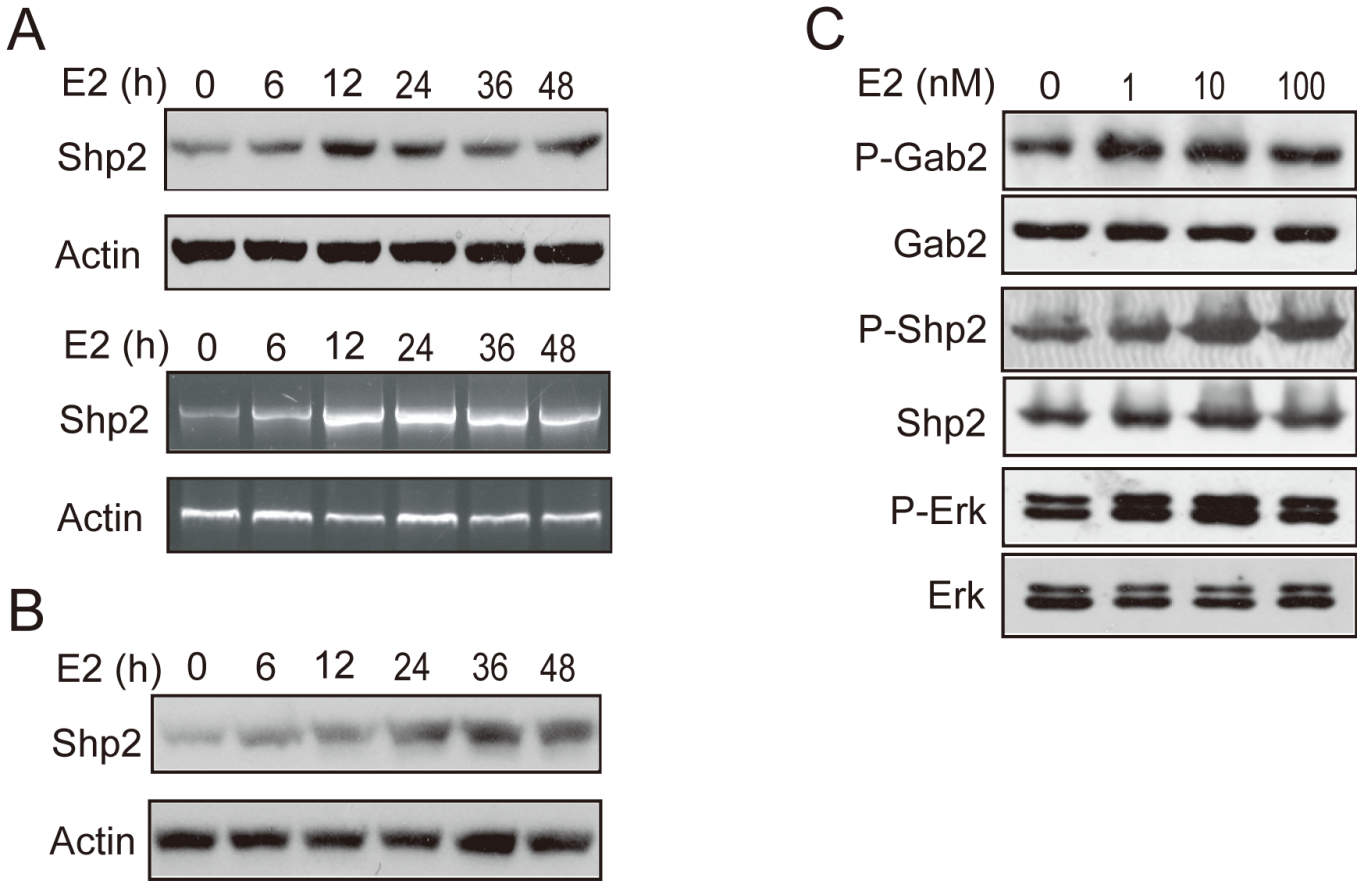
E2 induced the protein expression and phosphorylation of Shp2 and Gab2 in breast cancer cells. A: the protein and mRNA levels of Shp2 were shown in MCF7 cells treated with 10 nM E2 for different times. Actin served as the total protein control. B: Bcap 37 cells were treated with 10 nM E2, and Shp2 protein expression was checked by Western blotting. C: MCF7 cells were stimulated with different doses of E2 for 15 min, and then the phosphorylated protein of Shp2 or Gab2 was checked using the special antibody for active Shp2 or Gab2 (anti-phos-Shp2 or anti-phos-Gab2). Erk protein was also assessed as a total protein control, and the phospho-Erk level was recorded as an E2 activity control.

Previous studies and the current one showed that the Shp2 expression pattern was correlated with the protein level of ER in breast tumors [39], and that the expression of Gab2 (Shp2's pattern protein) was induced by E2 in breast cancer cells [40]. These results indicated that Shp2 may be involved in the estrogen action in breast cancer. To determine whether Shp2 expression was the answer to estrogen stimulation, we investigated the Shp2 protein level with its specific antibody in the ER positive cell lines MCF7 and Bcap37 after 17β estradiol (E2) treatment. The results showed that E2 stimulated the expression of Shp2 in a time-dependent manner (Fig 3A and B). Shp2 expression is clearly increased after E2 stimulation for 6 h, and then peaked at 12–36 h of treatment in both MCF7 (Fig 3A, upper panel) and Bcap37 (Fig 3B) cells. We also checked the mRNA level of Shp2 in MCF cells with E2-treatment with RT-PCR, and found E2 increased the gene transcription of Shp2 (Fig 3A, low panel).

To investigate whether estrogen activates Shp2 and Gab2, MCF7 cells were driven with different doses of E2 for 15 min. The phosphorylated protein level of Shp2 or Gab2 was assessed using the special antibody for phospho-Shp2 or phospho-Gab2 by Western blot analysis (Fig 3C). We found that E2 increased the protein of phosphorylated Shp2 (third panel from the top) or Gab2 (first panel from the top) at 1, 10 and 100 nM doses. The protein levels of Shp2 (fourth panel from the top) or Gab2 (second panel from the top) were unchanged.

### Shp2 interacts with cytoplasmic ER and then transmits the estrogen signal by activating Erk and Akt

**Figure 4:**
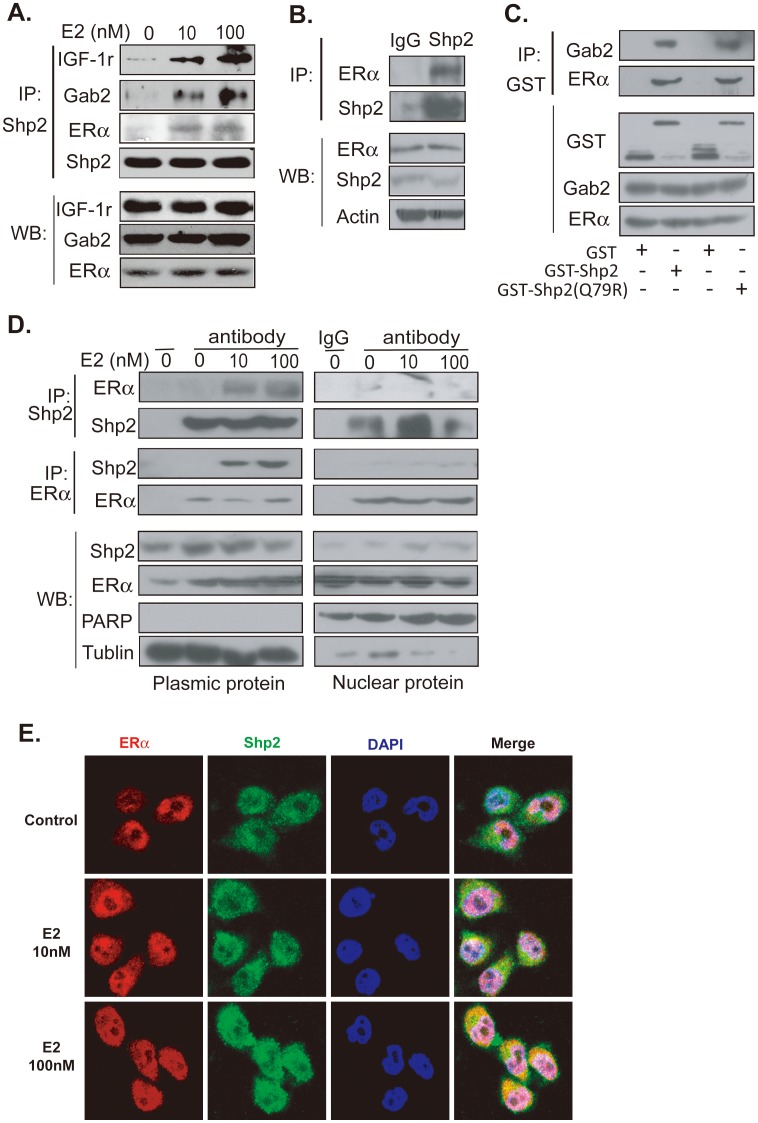
Shp2 has an interaction with cytoplasmic ERα undergoing E2 treatment. A: MCF7 cells were stimulated with 10 or 100 nM E2 for 15 min, and then the protein complex was pulled down using Shp2 antibody. The protein in this complex (top panel) or total cell lysate (bottom panel) was checked with Western blot analysis. B: The tissue lysate of mammary gland tumor in DMBA-induced tumor rat was incubated with antibody Shp2. Then the Shp2-containing protein complex was pulled down, and the ERα or Shp2 protein in this complex was checked with Western blot assay. C: MCF cells lysate was incubated with either GST alone or GST-Shp2 or GST-Shp2 (Q79R) protein. Then, Shp2–ERα–interactions were identified with GST pulldown assays. D: After the treatment of E2, MCF7 cells were broken. Then, the Shp2-containing protein complex in the cytoplasm or nucleus was pulled down by Shp2 or ERα antibody, respectively. The protein in this complex (top panel) or total cell lysate (bottom panel) was checked with Western blot analysis. PARP was regarded as a nuclear protein marker, while tublin was a marker of plasmic protein. E: MCF7 cells were stimulated with 10 or 100 nM E2 for 15 min, and then the protein location of ERα and Shp2 were checked by immunofluorescent staining with protein specific antibody.

The tyrosine phosphatase, Shp2, is a typical membrane receptor associated protein [20]. We therefore reasoned that Shp2 may mediate the estrogen signal by affiliating itself with the membrane receptor complex of estrogen. To test this hypothesis, MCF7 cells were treated with 10 nM or 100 nM E2 for 15 min, and an immunoprecipitation was performed with the antibody of Shp2 to determine whether Shp2 had bound with the ER containing complex. As expected, the protein of ERα or Gab2 was found in the complex pulled down by the Shp2 antibody in the cell lysate solution after the 10 nM E2 treatment (Fig 4A, top second and third panel, middle lane). In the control cells treated with ethanol, no band of ERα or Gab2 protein was found in the Shp2-containing complex (Fig 4A, top second and third panel, left lane). The combination among Shp2, ERα and Gab2 was stronger at a higher dose of E2 (100 nM) (Fig 4A, top second and third panel, right lane). The IGF-1 receptor, a membrane protein, was also found in this Shp2 containing complex (Fig 4A, top first panel). Moreover, both Shp2 and ERα were expressed in tumor tissue in DMBA-induced mammary gland tumor rat (Fig 4B, bottom panel). When protein complex was pulled down with Shp2 antibody, ERα was in this complex (Fig 4B, top panel, right band). Furthermore, GST tagged Shp2 protein was purified and incubated with MCF7 cell lysate. Then, the protein complex was pulled down with GST bead. The protein in this complex was checked using specific antibodies (Fig 4C). Results showed that ERα and Gab2 were pulled down by GST-Shp2 or GST-Shp2 (Q79R) (Fig 4C, top panel, the fourth band), an activity mutant Shp2. As a control, GST alone didn't pull down ERα (Fig 4C, top panel). These results indicated that Shp2 bound to the ER-containing complex in breast tumor cells.

To confirm whether Shp2 binds to ER receptor in extranuclear area, other two experiments were performed. Firstly, the interaction between Shp2 and ERα in the cytoplasm or nucleus was investigated (Fig 4D). After treated with E2, the cytoplasm or nucleus of MCF7 cell was separated. Here, PARP was used as a nuclear protein marker, while Tublin was regarded as a marker of plasmic protein. Fig 4D showed the cytoplasm was clearly separated from nucleus (Fig4D, bottom two panels). Then, the Shp2-containing protein complex in the cytoplasm or nucleus was pulled down by Shp2 or ERα antibody, respectively. When proteins in this complex were checked with specific antibody, ERα was found only in the Shp2-containing complex in the cytoplasmic solution of MCF7 cells treated with 10 nM or 100 nM E2 for 15 min (Fig 4D, the top left panel, the third and fourth band); Shp2 was also found in the ERα pulled down complex in the cytoplasmic solution rather than nuclear proteins (Fig 4D, the top third left panel, third and fourth band). Secondly, the protein location of ERα and Shp2 were checked by immunofluorescent staining (Fig 4E). Shp2 was expressed in the cytoplasm (Fig 4E, top panel, green color picture), while ERα was located in the nucleus (Fig 4E, top panel, red color picture) in MCF7 cells without E2 treatment. When cells were stimulated by 10 nM of E2 for 15 min, a part of ERα protein moved into the cytoplasm (Fig 4E, middle panel, red color) and merged with Shp2 protein (Fig 4E, middle panel, right picture, yellow color). When cells were treated with high concentration (100 nM) of E2, the movement of ERα and its merging with Shp2 was stronger (Fig 4E, bottom panel, red color); right picture, yellow color) Thus, these discoveries suggested that Shp2 may be bound to ER receptor around membrane.

**Figure 5:**
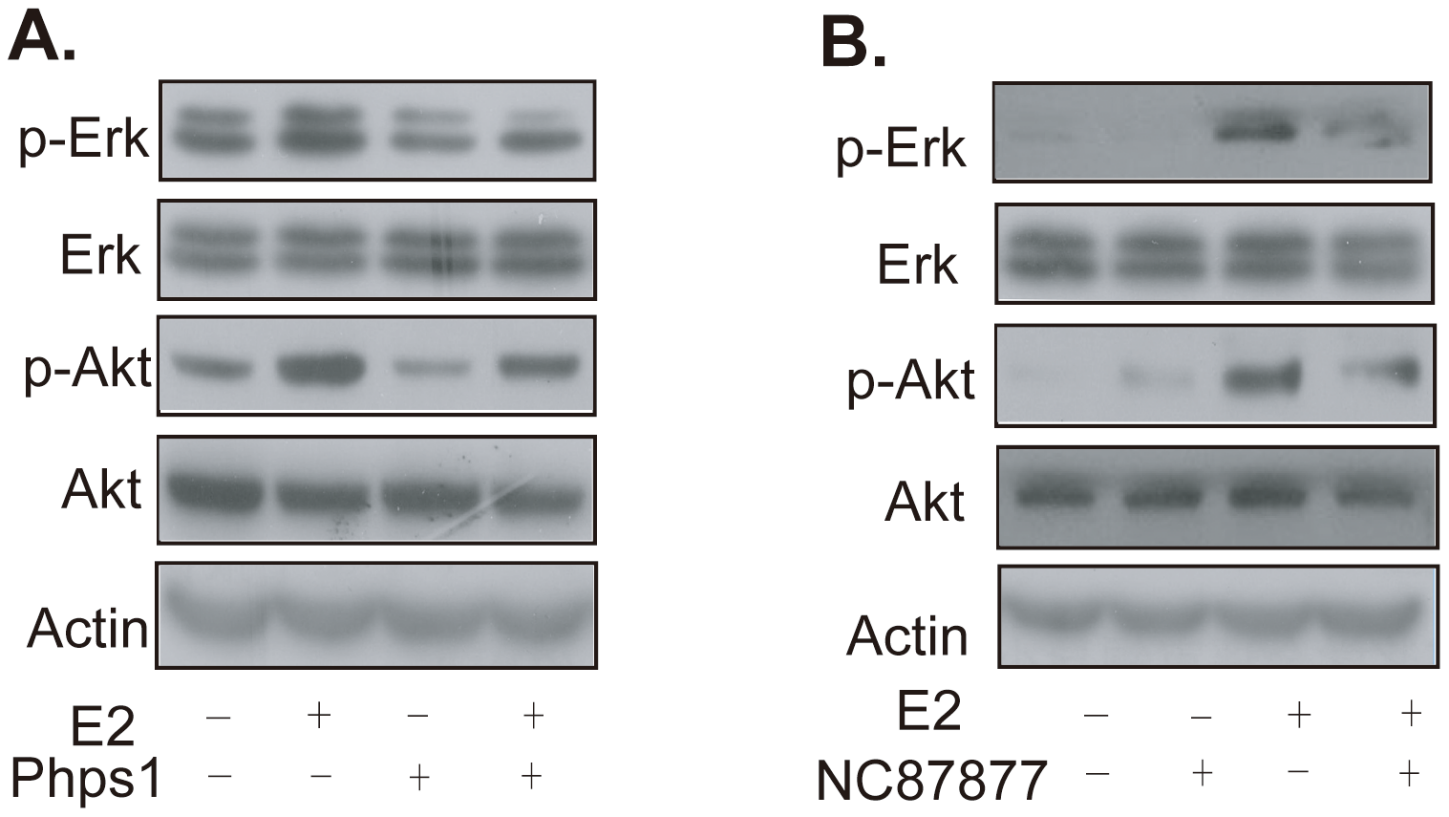
Shp2 mediated the E2-induced activation of cytoplasmic kinase Erk and Akt. MCF7 cells were pretreated with the Shp2 inhibitor phps1 or NSC87877 (a specific inhibitor of Shp2 and Shp1 activity) for 6 h and then treated with 10 nM E2. The phosphorylation of Erk or Akt was detected with specific antibodys. Protein levels of Erk, Akt and actin were also determined as the control total protein quantity.

MAPK and PKB/AKT cascades are two main pathways in the estrogen rapid signal transferred by extranuclear ER [14]. These pathways are further triggered by Shp2 in response to several growth factors. Therefore, we expected the mediation of the estrogen signal by Shp2 through the activation of the MAPK and PKB/AKT cascade pathways. To support this supposition, two specific inhibitors of Shp2 phosphatase activity, phps1 and NSC87877, were employed [41]. First, MCF7 cells were incubated with 10 µM phps1 or NSC87877 in serum-starved medium for 6 h, and then treated with 10 nM E2 for 15 min. DMSO served as mock control for the inhibitors. As shown in Fig 5, E2 markedly increased the protein level of phosphorylated Erk (top left panel, the second band; top right panel, the third band) and Akt (third left panel, the second band; third right panel, third band), but not total Erk (second left panel, second band; second right panel, third band) or Akt protein (the fourth left panel, the second band; fourth right panel, the third band). This finding reported that E2 triggered the activity of Erk and Akt after 15 min of stimulation (Fig 5). However, both phps1 and NSC87877 clearly blocked this E2-induced phosphorylation of Erk (top left panel, last band; top right panel, last band) and Akt (third left panel, last band; third right panel, last band), suggesting that Shp2 was an intermediate in E2 activation that triggered the Erk and Akt cascades.

### Shp2 mediates the bio-effects of E2 on downstream gene transcription

**Figure 6:**
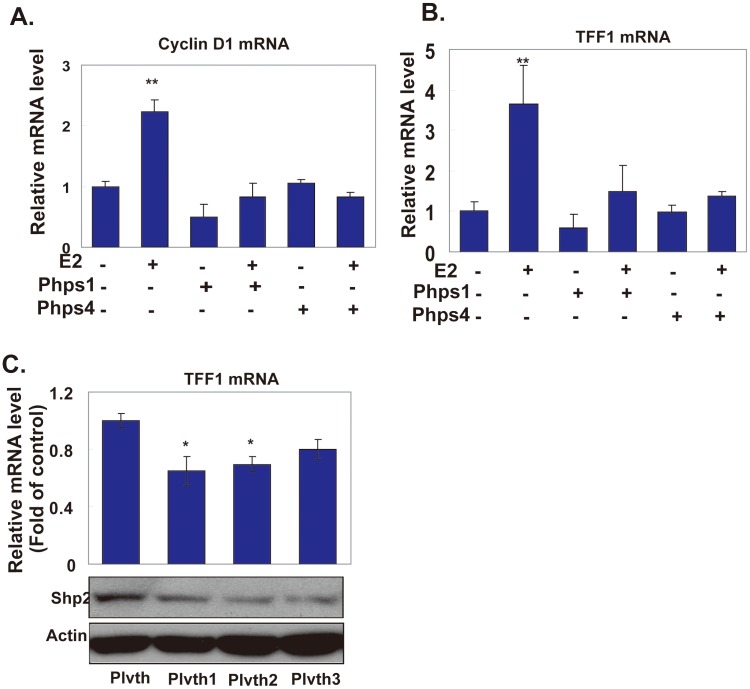
Shp2 was involved in the E2-induced gene transcription. A: The mRNA level of Cyclin D1 in MCF7 cells, which were pretreated with the Shp2 inhibitors phps1 or phps4 (a specific inhibitor of Shp2 activity) for 6 h and then stimulated with 10 nM E2 for 40 min. B: TFF1 mRNA level in MCF7 cells treated with E2 and Shp2 inhibitors for 18 h. C: E2-induced mRNA level in MCF7 cells transfected with the plasmids expressing Shp2 siRNA. The protein levels in each group of cells are shown in the bottom panel. The relative mRNA levels in all experiments were measured by real-time RT-PCR, and the relative value compared with the control group (the first column in the three figures) is shown. (* P<0.05, ** P<0.01) Results represent the mean and s.e.m. from three independent experiments.

To determine whether Shp2 mediates the E2-induced gene transcription, we selectively evaluated the effects of Shp2 inhibitors on the expression of estrogen downstream genes. Cyclin D1 is a key cell cycle regulator and also a downstream gene induced by estrogen through both rapid stimulation and long-term exposure [15]. We treated MCF7 cells with 10 nM E2 for 40 min and found that the mRNA level of Cyclin D1 was markedly increased to a level 2.5 fold higher than that in control cells (Fig 6A, the second column). But, the E2-induced expression of Cyclin D1 was remarkably abolished when cells were incubated with 10 µM phps1 or phps4 (Fig 6A, the fourth column or sixth column).

Trefoil factor 1 (TFF1) is an estrogen downstream gene that is directly induced by an estrogen signal. When MCF7 cells were treated with 10 nM E2 in combination with Shp2 inhibitors for 18 h, the E2 induced gene transcription of TFF1 was also inhibited (Fig 6B). In the absence of Shp2 inhibitors, E2 triggered a nearly fourfold higher expression of the TFF1 gene than in the control cells, whereas treatment with both phps1 and phps4 reduced the E2 stimulation to non-significant levels (Fig 6B). For further verification, we also examined the effects of E2 when the Shp2 protein level decreased in breast cancer cells using its specific small interference RNA (siRNA). MCF7 cells were transfected with three constructs that expressed siRNA of Shp2 (Plvth1, Plvth2 and Plvth3). After 48 h, the protein levels of Shp2 in MCF7 cells were clearly reduced by all three siRNAs (Fig 6C, middle panel). In these cells, E2-induced TFF1 expression was disrupted to different degrees (Fig 6C, top image), with a maximum suppression of about 30% (Fig 6C, top image, Plvth1 and Plvth2). Taken together, these results indicated that Shp2 was involved in the gene transcription of E2 responses.

### Shp2 promotes E2-stimulated cell proliferation in breast cancer cells

**Figure 7:**
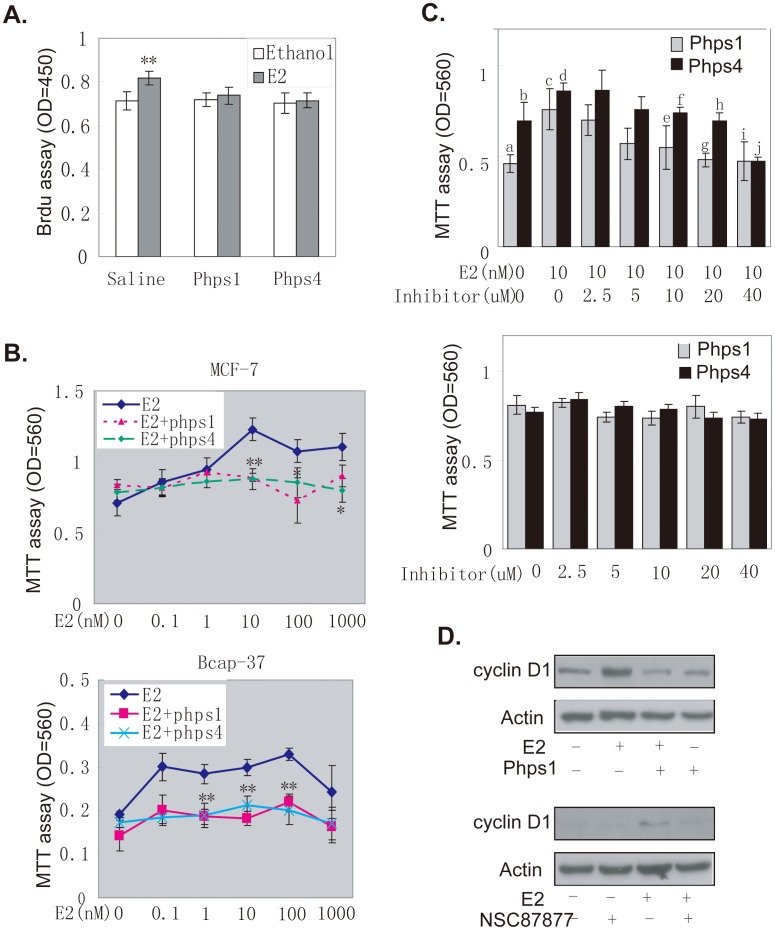
Shp2 mediated the E2-stimulated cell proliferation. A: The Brdu assay was used to determine E2-induced rapid DNA synthesis in MCF7 cells incubated with Shp2 inhibitors. The absorbance was read at 450 nm. (* P<0.05, ** P<0.01). B: MTT staining was performed to assess the cell growth of MCF7 (top panel) or Bcap37 (bottom panel) cells stimulated with different doses of E2, with or without Shp2 inhibitors phps1 or phps4 for 4 days. (* P<0.05, ** P<0.01). C: MCF7 cells were incubated with different doses of phps1 or phps4 (specific inhibitors of Shp2 activity), and treated with 10 nM E2 or ethanol for 4 days. Then, cell growth in the E2-treated cells (top panel) or control cells (bottom panel) was assayed by MTT staining. (d:b,e:c,f:d,h:d, P<0.05; c:a,g:c,i:c,j:d, P<0.01). D: The protein level of cyclin D1 or phosphorylated Akt in MCF7 cells with a long-term (18 h) to E2 and Shp2 inhibitors (phps1 or NSC87877). Actin protein served as the total protein control. The data in A,B and C represent the mean and s.e.m. from at least three independent experiments.

The main effects of estrogen in breast cancer are on cell proliferation [15]. We treated MCF7 cells with inhibitors of the Shp2 enzyme and then evaluated the effects of Shp2 on the E2-induced DNA synthesis by the bromodeoxyuridine (Brdu) assay. After a 30 min treatments, E2 (10 nM) increased DNA synthesis in the MCF7 cells by about 20% (Fig 7A, control). However, when cells were incubated with 10 µM phps1 or phps4, the same dosage of E2 had no effect on DNA synthesis (Fig 7A, phps1 and phps4). These results indicated that Shp2 was clearly associated with the estrogen rapid effects in breast cancer cells.

Using of the breast cancer cell lines MCF7 and Bcap37, we also evaluated the effects of Shp2 on E2-induced cell growth (Fig 7B). Briefly, MCF7 or Bcap37 cells were cultured in media with different doses of E2 and 10 µM Shp2 inhibitors. The media were changed daily. Cell growth was assayed by 3-r-2,5-diphenyltetrazolium bromide (MTT) staining after 4 d of treatment. E2 increased the cell growth of MCF7 or Bcap37 cells in a dose-dependent manner (Fig 7B, blue line), and cell proliferation was markedly enhanced from 10 nM to 100 nM E2 in MCF7 cells (Fig 7B, top panel, blue line) or 0.1 nM to 100 nM in Bcap37 cells (Fig 7B, bottom panel, blue line). However, this E2-induced cell growth was almost completely inhibited both in MCF7 and Bcap37 when cells were incubated with 10 µM phps1 (Fig 7B, purple line) or phps4 (Fig 7B, green line). When MCF7 cells were cultured in media with different doses of inhibitors and treated with 10 nM E2 for 4 days, both phps1 and phps4 showed a dose-dependent inhibition of E2-induced cell proliferation (Fig 7C, top panel). Significant inhibition was observed at 10 µM and became stronger with increased doses of phps1 or phps4 (20 and 40 µM) (Fig 7C, top panel).In the control, same doses of phps1 or phps4 did not affect the basic cell number without E2 treatment (Fig 7C, bottom panel).

For further confirmation, we investigated the effect of Shp2 inhibitors on the protein expression of Cyclin D1, a key regulator protein of cell proliferation. As shown in Fig 7D, the Cyclin D1 protein was induced and had a high level in MCF7 cells (top panel, second band; third panel, third band) when cells were treated with 10 nM E2 for 18 h. When cells were also treated with 10 µM phps1, the E2-induced protein expression of Cyclin D1 was also markedly decreased (top panel, fourth band). Similar treatment with 10 µM NSC87877 almost completely blocked the stimulatory effect of E2 on Cyclin D1 expression (third panel, last band). These results suggested that Shp2 mediated E2-stimulated cell proliferation in breast cancer cells.

## Discussion

The present research discovered that Shp2 mediation of the estrogen rapid extranuclear signal in breast cancer cells, and the suppression of Shp2 action blocked the development progress of the breast tumor. These results identified a novel associated protein of the extranuclear ER-initiated pathway, and suggested a novel mechanism for Shp2 action and considerably expanded the known biological effects of the extranuclear ER pathway in breast tumorigenesis.

We found that Shp2 was highly expressed in most of breast cancer cases (more than 60%), and also overexpressed in DMBA-induced rat mammary gland tumor. These findings confirmed those of previous studies [35], [39] and suggested that Shp2 may play a role in breast tumorigenesis. However, both our and previous results didn't find the significant correlation between Shp2 expression and clinical pathological states (such as Her2, ER, etc.) [39]. The possibly reason is that Shp2 was overexpression in most of the case of breast cancer. As a result, the correlation between Shp2 expression and clinical pathological states was covered. But, these results still suggested that Shp2 has a tendency to be overexpressed in ER-positive cases of breast cancer.

Based on DMBA-induced tumor or MMTV-pyvt transgenic tumor animal model, our results showed that the inhibition of Shp2 activity delayed tumor formation and blocked tumor growth. These results confirmed those of previous studies [35], [38]. A study on a transgenic animal model or three-dimension cell cultures with Gab2 deletion has indirectly shown that Shp2 is involved in breast epithelial neoplasia and metastasis [35]. Recently, Shp2 was directly demonstrated to promote the proliferation and invasion of breast epithelial cells in a three-dimensional cell culture, as well as enhance the metastasis in a xenograft mice model [38]. These results provided solid evidence of the role of Shp2 in breast tumorigenesis in vivo. DMBA-induced breast tumor is hormone dependent [42], [43], which indicated that Shp2 may be involved in hormone regulation on tumorigenesis in mammary gland.

The present study found that Shp2 formed a complex with receptors of estrogen near cytoplasmic membrane responding to estrogen stimulation, and then transduced the estrogen signal by triggering the cytoplasmic pathways ERK and AKT to induce gene transcription (such as Cyclin D1 and TFF1), DNA synthesis, and cell proliferation in breast cancer cells. The exactly mechanisms of how the estrogen receptor protein moved into the cytoplasm and combined with Shp2 when treated with E2 is not clear [12]. Shp2 may form a complex with ERα by receptors of growth factors (such as IGF-1) [12]. Another, Shp2 also may directly bind to ER through its SH2 domain, because tyrosine phosphorylation sites have been found in ERα protein [44]–[46]. Based on that Shp2 is a membrane receptor associated protein, Shp2 may form a complex with ER and membrane receptors. In our research, interaction between Shp2 and ER was not found in nucleus, although Shp2 protein was also found in nucleus. Recently, our collaborator also found that Shp2 associated with ER integrated leptin and estrogen signals in forebrain neurons [47], which confirmed our discovery. So, these results suggested that Shp2 may be a novel extranuclear ER-associated protein, which affiliate ER to trigger downstream kinase cascades to mediate genomic or non-genomic effects of estrogen. Membrane receptor-associated proteins have been found to play crucial roles in extranuclear ER initiated signal. Our finding on new ER-associated protein will promote the understanding of the action of estrogen, especially in breast tumorigenesis.

Shp2 may be also involved in the effects of estrogen on the late stages of cancer development, particularly on drug resistance and metastasis. Endogenetic growth factors (notably EGF and IGF-1) and the Her2/neu signaling pathway play crucial roles in the progress of metastasis and drug resistance [17], [48]. The extranuclear ER initiated pathway is well known to activate these signals [49], although the underlying mechanism involved in the crosslink between the mER pathway and growth factors has not yet been revealed [50]. Shp2 was found to mediate an extranuclear ER-initiated pathway and to associate growth factor signals by triggering a cytoplasmic cascade [20]. Thus, Shp2 was involved in the crosslink between estrogen and growth factors, and also promoted the development of breast carcinogenesis. Besides, an increasing number of studies reported that estrogen signal induced the expression of metastasis-related genes, such as AGR2 [51], Gab2 [40], LMO4 (Lim only protein 4) [52], etc. These protein expressions in breast tumors have been found to be highly correlated with poor prognosis and low survival. These genes also independently promote the progression of metastasis in breast tumors [35], [40], [51]. Shp2 has been proven to enhance cell migration in MCF7 cells and promote the metastatic development of breast tumors in an animal model [35]–[38]. Thus, Shp2 may also be an estrogen induced metastasis gene and independently promote metastasis progression in breast tumors even when the estrogen signal has disappeared.

In conclusion, these results introduced a new mechanism for Shp2 oncogenic action and shed new light on extranuclear ER-initiated action in breast tumorigenesis by identifying a novel associated protein, Shp2, for this pathway, which might benefit the therapy of breast cancer.

## Materials and Methods

### Cell culture and reagent

Breast cancer cell lines MCF7 and Bcap37 were purchased from the Type Culture Collection of the Chinese Academy of Sciences, Shanghai, China. Both cell lines were ER positive. Cells were grown in normal DMEM with 10% FBS, 100 unit/mL penicillin and 100 µg/mL streptomycin. Prior to all experiments, cells were pretreated for at least 1 week with an estrogen-free medium (phenol red-free DMEM with 10% dextran-coated charcoal serum, 100 units/mL penicillin, 100 µg/mL streptomycin, 0.5 mM sodium pyruvate, and 2 mM l-glutamine) to prevent the effects of serum-derived estrogenic compounds. Cell transfection was carried out using Lipofectamine 2000 (Cat. 11668, Invitrogen) according to the manufacturer's instructions.

E2 (E8875, Sigma) was prepared as a 10 mM solution with ethanol and stored at −80°C. Tamoxifen (T5648, Sigma) or toremifene (T7204, Sigma) was dissolved in 10 mM DMSO. Phps1 (P0039, Sigma,) was dissolved in PBS solution. NSC87877 (565851, Millipore) and phps4 (a gift from Dr. Yuehai Ke (Department of Medicine, Zhejiang University) were dissolved in DMSO. Plasmids expressing siRNAs of Shp2 Plvth-H1, Plvth-H2, and Plvth-H3, as well as a control plasmid Plvth were also prepared as previously described [53]. Unless otherwise indicated, all other chemicals were obtained from Sigma/Fisher.

### Breast cancer samples and immunohistochemical staining

200 Primary breast cancer samples were collected from the Department of Pathology, Fujian Provincial Tumor Hospital between September 2007 and July 2009. The study protocol was approved by the Research Ethics Committee of the Fujian Provincial Tumor Hospital (200710), and written informed consents were obtained from all participants. No patients had received chemotherapy, hormone, radiation, or immunotherapy before surgery. The diagnosis for each case was histopathologically confirmed and clinicopathological data were obtained after histopathological examination. Excluding the case missing some information, we obtained 151 cases which had accurate clinical and pathological information.

The sections were immunoassayed with primary antibodies for Shp2 (Santa Cruz Biotechnology, Inc., 1∶2000) and then incubated overnight at 4°C in a humidified chamber. The slides were reacted with the secondary antibodies for horse anti-mouse IgG (Beijing Zhongshan Jinqiao Co. Ltd., P.R. China) for 0.5 h at room temperature. Afterwards, the sections were incubated with diaminobenzidine hydrochloride for 5 min, washed with PBS, and counterstained with hematoxylin. Control slides without primary antibody were examined in all cases. Shp2 was expressed in the cells stained brown. (+) means a mild expression of Shp2, while ++ or +++ represents a strong expression of Shp2.

### Animal treatment

For the DMBA-induced mammary gland tumor model, 5–6 week-old adult female Sprague–Dawley rats were obtained from Shanghai Breeding Co. Ltd., P.R. China. The rats were housed under standard conditions of humidity, temperature (25±2°C), and light (12 h light/dark) at the Animal Research Centre, Xiamen University. After 1 week of breeding, the animals were randomly divided into two groups. One group (n = 60) was fed a single intragastric dose of DMBA (Sigma Chemical Co, St. Louis, MO, USA) in olive oil (100 mg/kg body weight). As the control group, the remaining rats (n = 8) only received olive oil. After approximately 4 weeks, the DMBA-treated rats were randomly assigned to two groups. One group (n = 30) was treated with a subcutaneous injection of phps1 (Sigma Chemical Co, St. Louis, MO, USA) in saline solution (0.5 mg/kg body weight/day) for seven continuous days. The other control group (n = 30) was injected with saline only. The animals were palpated twice weekly to detect tumors beginning from 6 weeks after DMBA administration.

For the transgenic mammary gland tumor model, 6 week-old female MMTV-pyvt mice were randomly divided into two groups. Test mice (n = 30) were treated with a subcutaneous injection of phps1 (Sigma Chemical Co, St. Louis, MO, USA) in saline solution (0.5 mg/kg body weight/day) for seven continuous days. Control mice (n = 30) were treated with saline only. The mice were palpated twice weekly to detect tumors.

All experimental procedures were approved by the Animal Welfare Committee of Research Organization (X200703), Xiamen University.

### Co-immunoprecipitation, Western blot analyses and GST pull-down assays

Co-immunoprecipitation analyses of whole cells lysate or Cytoplasmic/nuclear protein solution were performed as previously described [54] using Shp2 antibody [55] to pull down the protein complex. Protein levels were determined by Western blot analysis. The antibodies to ERα (mc20, SC-542), IGF-1R (3B4, SC-462), Cyclin D1 (sc-753), Akt1 (B-1, SC-5298), p-Akt (Ser 473) (SC-798512), Erk (C-16, SC-16982), p-Erk (Thr 202/Tyr 204) (SC-16982), and β-Actin (C4, SC-47778) were purchased from Santa Cruz Company. The other antibodies were as follows: Gab2 (06-967, Millipore), Phospho-SHP-2 (Tyr580) (Cell Signal 3703), Phospho-Gab2 (Tyr452) (Cell signal 3882)

The GST pull-down assays were performed as previously described [54]. The plasmid pGEX4T1-Shp2 (a gift from Dr. Gensheng Feng at the University of California San Diego) was transformed into the engineering strain BL-21 and the GST-tagged Shp2 protein was expressed. Then, GST protein or GST-Shp2 fusion protein was incubated with MCF cell lysate at room temperature for 3 hours. After washing three times, the glutathione–agarose beads were resuspended in SDS sample buffer, and the protein interaction was analyzed with specific antibody.

### Nuclear and cytoplasmic protein preparation

The nuclear and cytoplasmic protein extraction of MCF7 cells was prepared using NE-PER Nuclear and Cytoplasmic Extraction Kit (Cat. 78833, Thermo) following the protocol provided by manufacture. In briefly, cells were harvested and washed with cold PBS two times. Then, about 5×10^6^ cell were transferred into a 1.5 ml tubes and pelleted by centrifugation at 500g for 2 min. The supernatant was discarded as completely as possible. The dry pellet was full suspended with 400 µl CER I solution (appropriate volume should be 10 times of the cell pellet volume) by vigorously vortexing for 15 seconds, and then stored on ice for 10 minutes. 22 µl cold CER II was added in the tube. After mixing by vortex for 5 seconds, the solution was incubated on ice for 1 minute. The supernatant (cytoplasmic extract) was obtained by a maximum speed centrifugation for 5 minutes. The pellet was suspended again with 200 µl cold NER solution by vigorously vortexing for 15 seconds and stored on ice for 10 minute. Repeat these steps for another three times. At last, the nuclear extraction was collected by centrifuging at a maximum speed 6,000 rpm for 10 minutes. The volume ratio of CER i:CER II: NER reagents should be maintained at 200:11:100, respectively. Tublin (#2125) and PARP (#9532) antibody were purchased from Cell Signal.

### Immunofluorescent staining

For immunofluorescent staining, MCF7 cells were seeded on clean glass cover slips. After treated with E2, cells were stained following immunofluorescent protocol. In brief, cells were fixed with with 4% formaldehyde, permeabilized with 0.2% Triton X-100 and blocked with 5% normal serum. Then, cells were incubated with specific primary antibody for 1 h. At last, an appropriate fluorophore-conjugated secondary antibody was applied. The antibodies were as follows: ERα (04-227, Millipore), DAPI (D9564,Sigma), Rabbit FITC (ZF-0311,Zhongshan jinqiao, Beijing, China), and mouse Alexafluor 488 (ZF-0512,Zhongshan jinqiao, Beijing, China).

### MTT cell growth assays

Cell growth was assessed by the MTT assay using a colorimetric (MTT) kit (CT-01, Millipore). The cells were seeded at a density of 2000 cells/well in 96-well plates, and then treated with E2 or Shp2 inhibitor at the desired dose. After growing for 4 days, the cell number was determined by MTT staining. In a typical determination, cells from 10 wells were incubated with MTT (62.5 µg/well) for 4 h. Cellular MTT was solubilized with acidic isopropanol, and the absorbance was measured at 560 nm using a Multiscan plate reader (MK3, Thermo).

### Real-time PCR

Total RNA was extracted from cells using TRIzol reagent (Invitrogen), and complementary DNA was synthesized with 5 µg of total RNA using a Revert Aid First Strand cDNA Synthesis Kit (Fermentas). Real-time PCR was performed using SYBR GreenER Qpcr SuperMix (Invitrogen) and the ABI Prism 7900HT platform (Applied Biosystems) following standard protocols from the supplier to the determine the threshold cycle (Ct). GAPDH was used as an endogenous control. Relative quantification was performed using the Prism Sequence Detection software (Applied Biosystems). The primer sequences were as follows: sense 5′-TTT GGA GCA GAG AGG AGG-3′, antisense 5′-TTG AGT AGT CAA AGT CAG AGC AG-3′ (**TFF1**); sense 5′-TGG TCA CCA GGG CTG CTT-3′, antisense 5′-AGC TTC CCG TTC TCA GCC TT-3 (**GAPDH**); and sense: 5′-CTG GCC ATG AAC TAC CTG GA-3′, antisense 5′-GTC ACA CTT GAT CAC TCT GG-3′ (**Cyclin D1**); sense 5′-ATG ACA TCG CGG AGA TGG TT-3′,antisense 5′-TCA TCT GAA ACT TTT CTG CTG T-3 (ptpn11/Shp2); sense 5′-CTT CAA CAC CCC AGC CAT GTA-3′, antisense 5′-TAG AAG CAT TTG CGG TGG ACG-3 (β-actin)

### DNA synthesis assay

E2-induced rapid DNA synthesis in MCF7 cells was assessed by the incorporation of Brdu into a newly synthesized DNA using a Brdu Cell Proliferation Assay kit (Cat. 2750, Chemicon). Cells were plated in 96-well plates at a density of 5000 cells/well. On the second day, cells were incubated in serum-free medium overnight and pretreated with 10 µM phps1 or phps4 for 4 h. Brdu was then added to the serum-free medium with fresh phps1 or phps4, and cells were incubated for 3 h. Lastly, 10 nM E2 or ethanol was added to the medium and the cells were treated for another 30 min. The Brdu label was analyzed following the manufacturer's protocol for reading absorbance at 450 nm using a Multiscan plate reader (MK3, Thermo).

### Statistical analysis

The values for all experiments were plotted as the mean ± SD from at least three repetitions. Three independent experiments were performed for each assay. The statistical significance of differences between groups was analyzed by Student's t test or ANOVA. P<0.05 was considered significant.

## Supporting Information

Table S1 **The correlation between Shp2 expression level and clinical or pathological progress indicators.**
(DOC)
