# Supplementary material for: Tyrosine Phosphatase Shp2 Mediates the Estrogen Biological Action in Breast Cancer via Interaction with the Estrogen Extranuclear Receptor
Source: PLoS One. 2014 Jul 21;9(7):e102847. doi: 10.1371/journal.pone.0102847 (PMC4105620; doi:10.1371/journal.pone.0102847)
Supplement: Table S1 — The correlation between Shp2 expression level and clinical or pathological progress indicators. (DOC) [file pone.0102847.s001.doc]

Supplemental Table S1. The correlation between Shp2 expression level and clinical or pathological progress indicators

| Item | Shp2 expression* | P value |
| --- | --- | --- |
|  | Low(-/+) high(++/+++) |  |
| Age(year)  <55  ≥55  Tumor size  <2  ≥2  Lymph Node  Negative  Positive  ER  Positive  Negative  PR  Positive  Negative  ErBb-2  Positive  Negative  Histological grade  I-II  III-IV  TNM stage  I-II  III-IV | 50 57  10 24  8 15  52 76  32 48  28 40  40 70  20 21  30 57  30 34  27 55  33 36  36 60  24 31  48 82  12 9 | 0.114  0.6505  1.000  0.112  0.1336  0.0686  0.4925  0.0949 |

* the protein expression level of Shp2 in breast cancer samples was determined by immunostaining with anti-Shp2. Shp2 was expressed in the cells stained brown. (+) means a mild expression of Shp2, while ++ or +++ represents a strong expression of Shp2.
